# Call for consensus debate on mobile phone radiation and health: Are current safety guidelines sufficient to protect everyone's health?

**DOI:** 10.3389/fpubh.2022.1085821

**Published:** 2022-12-15

**Authors:** Dariusz Leszczynski

**Affiliations:** ^1^Biological and Environmental Sciences, University of Helsinki, Helsinki, Finland; ^2^Frontiers, Lausanne, Switzerland

**Keywords:** consensus, wireless communication, RF-EMF, safety guidelines, health effects

The current deployment of the fifth generation of wireless communication technology (5G) has reignited the long-standing debate around the possibility of health effects from the radiation emitted by the existing wireless communication devices and networks and the new ones introduced by the 5G. The opposition of the part of society toward wireless communication technologies, including 5G, is caused by the uncertainty of whether radiation emitted by wireless devices and networks affects human health and the health of the environment's fauna and flora. Furthermore, a sizable part of the population considers themselves to be sensitive to wireless radiation (1, 2). According to the definition of health by the World Health Organization, this is by itself a health effect of the radiation emitted by wireless technology (3).

When reviewing published science, there are often heard claims, from some scientists and the telecommunication industry, that the topic of the radiofrequency modulated electromagnetic fields (RF-EMFs) emitted by wireless communication devices and networks has been very thoroughly researched and that there are available *thousands and thousands* of studies available on the health effects of RF-EMF.

Such claims are inaccurate and misleading.

A specialized database in Germany, the EMF Portal^1^, has collected 37,104 publications of all types of studies on various frequencies of EMFs. Of these, there are 1,951 studies concerning specifically wireless communication's RF-EMF, and only 449 studies are on 5G (as of 16 November 2022).

This limited number of studies that examined the biological and health effects of RF-EMF is being interpreted either as evidence of a lack of health harm or evidence of health harm.

The evaluations of the same scientific evidence come to different conclusions depending on the scientists performing the analysis.

Evaluations of the research conducted by two groups of scientists, forming the International Commission on Non-Ionizing Radiation Protection (ICNIRP)^2^ and the International Committee on Electromagnetic Safety of the Institute of Electrical and Electronics Engineers (IEEE-ICES)^3^, are used to set international safety guidelines. Both ICNIRP and IEEE-ICES claim that scientific evidence shows a lack of harmful health effects. The opinion of ICNIRP is, historically already, recommended by the World Health Organization (WHO), and because of it, these WHO recommendations are also followed by the telecommunication industry and the majority of the national governments.

However, the evaluation of the same scientific evidence by other teams of scientists including the BioInitiative^4^, the International Committee on Electromagnetic Safety (ICEMS)^5^, or the recently established International Commission on Biological Effects of the Electromagnetic fields (ICBE-EMFs)^6^ leads to conclusions that the scientific evidence shows definite harm to health.

There are significant management differences between ICNIRP/IEEE-ICES and BioInitiative/ICEMS/ICBE-EMF. There are also significant differences in how scientific studies are qualified and evaluated and how the final conclusions are drawn. These differences influence how the opinions of these organizations are considered scientifically valuable.

As a result, the opinions of scientists of BioInitiative and ICEMS are being largely dismissed by the WHO, telecommunications industry, and governments, as of insufficient quality and incorrect in their conclusions (the opinion of the ICBE-EMF has been published just recently and there are yet no comments on it from the WHO, telecoms, or governments). Hence, to be heard by the national radiation safety authorities and governments, scientists of these organizations and general public activists have begun to go to courts of law to prove that their interpretation of scientific evidence is correct [e.g., refer footnote^7^].

Here are the three examples of opposing conclusions, generated by the evaluation of the same scientific evidence:

- In 2020, ICNIRP published updated guidelines for the protection of the public from the effects of exposure to man-made EMFs (4) where it concluded that,

“*The only substantiated adverse health effects caused by exposure to radiofrequency EMFs are nerve stimulation, changes in the permeability of cell membranes, and effects due to temperature elevation. There is no evidence of adverse health effects at exposure levels below the restriction levels in the ICNIRP (1998) guidelines and no evidence of an interaction mechanism that would predict that adverse health effects could occur due to radiofrequency EMF exposure below those restriction levels*”

- In 2022, the BioInitiative published an update^8^ to their 2019 report that contains recommendations on the protection of people from the effects of exposure to man-made EMFs where it concluded that,

“*Bioeffects are clearly established at very low levels of exposure to electromagnetic fields and radiofrequency radiation. Bioeffects can occur in the first few minutes at levels associated with cell and cordless phone use. Bioeffects can also occur from just minutes of exposure to mobile phone masts (cell towers), WI-FI, and wireless utility ‘smart' meters that produce whole-body exposure. Chronic base station level exposures can result in illness*. […] *Bioeffects with chronic exposures can reasonably be presumed to result in adverse health effects…*”

- In 2022, the ICBE-EMF published an extensive commentary (5) on the health effects of RF-EMF exposures and the validity of the safety guidelines and concluded that,

“*25 years of extensive research on RFR demonstrates that the assumptions underlying the FCC's and ICNIRP's exposure limits are invalid and continue to present a public health harm. Adverse effects observed at exposures below the assumed threshold SAR include non-thermal induction of reactive oxygen species, DNA damage, cardiomyopathy, carcinogenicity, sperm damage, and neurological effects, including electromagnetic hypersensitivity. Also, multiple human studies have found statistically significant associations between RFR exposure and increased brain and thyroid cancer risk*.”

The differences in the evaluation of scientific evidence by ICNIRP, IEEE-ICES, BioInitiative, ICEMS, and ICBE-EMF groups are caused by the participating scientists. Namely, each of these groups self-selects member scientists. A close look at the composition of each of these groups of scientists clearly shows that each of these groups selects only scientists with the same opinion on the issue of RF-EMF and health. Hence, there is easily achievable internal consensus within each of these groups. ICNIRP or IEEE-ICES groups, by selecting scientists who consider that there is no evidence of harm caused by RF-EMF exposures, arrive at a consensus opinion that RF-EMF is safe when manufacturers and users follow ICNIRP/IEEE-ICES safety guidelines. In contrast, BioInitiative, ICEMS, and ICBE-EMF groups, by selecting scientists who consider that there is evidence of RF-EMF harming health, arrive at a consensus opinion that RF-EMF is not safe when the user follows current safety guidelines. Hence, these groups advocate lowering RF-EMF exposures and implementing precautionary measures or precautionary principles, as defined by the European Union^9^. This way of self-selecting members, scientists with certain opinions, leads to and perpetuates the polarization of the view on the causality link between RF-EMF exposures and human health.

Primarily, these groups of scientists have not only different views on the meaning of the scientific evidence but also differing views on what exposures are safe and unsafe. The safety guidelines proposed by these groups of scientists differ. Therefore, the scientifically legitimate question is to ask whether the currently used safety guidelines developed by ICNIRP/IEEE-ICES and recommended worldwide by the WHO are sufficiently protecting users or should the safety guidelines be revised as proposed by ICBE-EMF or BioInitiative. This is the question to which those concerned with RF-EMF exposures would like to get a clear answer. This is also in the interest of governments and industry, to become assured by the scientific consensus that the guidelines are indeed correct. Having guidelines set by a same-minded group of scientists might not be sufficiently assuring.

There was only one scientific evaluation of RF-EMF studies where the gathered group of scientists represented a full spectrum of diverse scientific opinions on RF-EMF and health, cancer in particular. This diverse group of scientists gathered in May/June 2011 at the Headquarters of the International Agency for Research on Cancer (IARC) in Lyon and following intense debates came up with a recommendation that RF-EMF is a possible human carcinogen.

- Scientists invited to the working group of IARC concluded (6) that,

“*Given the limited evidence in humans and experimental animals, the Working Group classified RF-EMF as “possibly carcinogenic to humans” (Group 2B). This evaluation was supported by a large majority of Working Group members*.”

The opinion of the International Agency for Research on Cancer is in disagreement with the opinions of ICNIRP, IEEE-ICES, BioInitiative, and ICEMS (ICBE-EMF did not exist yet in 2011). ICNIRP and IEEE-ICES consider that there is no evidence that RF-EMF is carcinogenic. BioInitiative, ICEMS, and ICBE-EMF consider that the evidence is sufficient to classify RF-EMF as a human carcinogen. The 2011 IARC classification was the middle way when it considered RF-EMF to be a possible human carcinogen (Group 2B on the IARC scale). As new studies were published after IARC classification in 2011, in 2019, an IARC Advisory Group to Recommend Priorities for the IARC Monographs during 2020–2024 met in Lyon, and it recommended that the carcinogenicity of the RF-EMF should be re-evaluated within the next 5 years (high priority)^10^.

Scientific debates at IARC Headquarters in Lyon in 2011 showed that there is no such thing as scientific consensus support for either the opinions presented by ICNIRP/IEEE-ICES or BioInitiative/ICEMS/ICBE-EMF.

However, the telecommunication industry is concerned with the polarization of scientific opinions on RF-EMF and health. Recently, on 11 October 2022, the GSM Association (GSMA), an umbrella organization representing operators of mobile networks, held the 11th GSMA EMF Forum where one of the discussion sessions was dedicated to the question “Is there a consensus among scientific reviews of RF-EMF health risks?”^11^. Unfortunately, GSMA invited solely speakers from one end of the opinions spectrum, scientists with the same opinions as those of ICNIRP and the WHO (11)^11^. This is not the best way to establish whether or not consensus exists when only one side of the debate is invited.

The diversity of interpretations of RF-EMF science reflects a broader problem of RF-EMF research. When the results of experimental studies are difficult to interpret, and the outcomes of studies are mostly ambiguous, it is up to individual scientists and groups of scientists to determine the significance of the results of such studies. Scientists who are more worried about the possible health effects will provide a different final evaluation of the ambiguous science than the scientists who are less worried about the possible effects.

Research on RF-EMF and health are being conducted for a long time, but there are still significant gaps in the knowledge. There is a lack of studies where, as far as it is ethically permissible, the *in vitro* and *in vivo* discovered biological effects would be examined for their occurrence and physiological strength in humans. For example, *in vitro* and *in vivo* studies have indicated that RF-EMF exposures might affect such cellular processes as a classic stress response (so-called *heat shock stress*), oxidative stress response, or DNA integrity. Without human volunteer studies, showing that such biological responses occur with strength sufficient to alter the normal physiology of mobile phone users, it is questionable to claim that human health is affected by RF-EMF exposures, no matter whether the exposures meet, or not, the current safety guidelines.

There is also an often expressed opinion that the majority of the RF-EMF studies are of poor quality, have too small a sample size for reliable statistics, and provide *in vitro* and *in vivo* evidence that has not been proven to occur in living humans. The most recent critical reviews showing the low quality of science have concerned the 5G technology and health (7–9). Hence, there is advocated a strong and urgent need for better-quality research (10).

Despite the general agreement that the currently available scientific evidence is of poor quality and that there are significant gaps in the knowledge, this *poor and inadequate* scientific evidence is being used to claim that there is either no evidence of harm or that evidence of harm has been established. Such statements not only lack logic but also are morally and ethically questionable. If the scientific evidence used either to support claims of safety, or lack of it, is of poor scientific quality, then claims of safety, or lack of it, are unreliable because they lack solid support from quality scientific studies.

This impasse in scientific interpretation has been ongoing for several years. The severely polarized debate on RF-EMF and health causes conspiracy theories to be born. It is possible that some of the scientific evidence might be over-interpreted or under-interpreted by the different teams of scientists. The reasonable way to resolve the problem of RF-EMF and health would be to find a common interpretation of science by gathering scientists from both sides of the debate.

The starting point, to suppress conspiracy theories and unwanted disinformation, is to openly debate the validity of the science review performed by ICNIRP, IEEE-ICES, BioInitiative, ICEMS, and ICBE-EMF.

Industry umbrella organizations, such as GSMA and Mobile and Wireless Forum (MWF), should consider the consequences of potentially possible incorrect scientific opinions provided by ICNIRP/IEEE-ICES that are recommended by the WHO and closely followed by the telecom industry. For example, members of ICNIRP do not have any legal responsibility for their opinions, but the telecom industry which uses ICNIRP-recommended safety guidelines has legal responsibility if the telecom-produced devices would be demonstrated to cause health harm.

In 2012, I called for a round-table debate on what means the scientific evidence that was gathered in biomedical research on the effects of radiation emitted by wireless communication devices and networks (RF-EMF)^12^. The round-table initiative was proposed to determine whether there is any possible common ground in the evaluation of the meaning of the scientific evidence and to ensure that the safety guidelines are correct. Unfortunately, in 2012, neither ICNIRP nor BioInitiative was interested in the round-table debate. In their e-mails/letters sent to me, both ICNIRP^13^ and BioInitiative^14^ have strictly opposed any round-table debate with each other. It has been 10 years since 2012, and the polarized situation remains the same in 2022. The scientific evidence is reviewed and evaluated in two different, often opposing ways by two sets of scientists that do not overlap.

In conclusion, I recommend conveying a round-table debate that would assess the current status of the science on RF-EMF and health and would review the adequacy of the current safety guidelines. The round-table debate might not change the current *status quo*. However, in the current situation, where there are significant gaps in knowledge and current studies are widely regarded as of poor quality, it would be reassuring if scientists from this highly polarized research field would come together and engage in a meaningful debate.

The pre-requisite for the debate should be *crystal-clear* transparency of the whole process and open-mindedness of all participants, irrespective of their current opinions or alliances.

The major questions to be answered are where the debate would be conducted, who would be the participants, how the debate would be conveyed, and who would finance it. Here are a few crude suggestions:

- Where: One possible place is the WHO in Geneva, but it is likely that some scientists (ICBE-EMF, ICEMS, and BioInitiative) would very strongly oppose this location as favoring the ICNIRP/IEEE-ICES. Another possible site is the IARC in Lyon. IARC is an institution with a long history of performing evaluations of cancer science. In this particular case, the evaluation should be expanded to include not only cancer but also all possible health effects.
- Who shall participate: Each of the two sides of the debate could select its own group of scientists for the debate. There could also be, amicably, a group of independent scientists who are experts in health risk evaluation, epidemiology, animal studies, and laboratory *in vitro* studies but who are never involved in RF-EMF research to bring an additional *scientific angle* to the debate. The total number of debating scientists should, preferably, not exceed 30 (+ a chairperson), which is a group size suitable to facilitate efficient scientific debate. Furthermore, it would be important for the scientific debate that each scientist would represent solely his own expertise and not be considered or acting as a representative of any organization.
- How the debate shall be conveyed: Evaluation of the science could use the protocols developed and used by IARC^15^^,^^16^, expanded to include all health effect studies.
- Financing: Assuming that the IARC would provide facilities and that the scientists would provide their expertise and time *pro publico bono* free of charge, the only costs to cover would be for travel and lodging. For such an important issue, concerning billions of users and non-users of wireless technology, one could expect that grant from the United Nations or WHO or any global institution would be possible to obtain to cover the travel of experts.

In my opinion, the controversy surrounding the possibility of health effects from exposure to RF-EMF emitted by wireless communication devices and networks will very likely continue as long as the two opposing teams of scientists do not discuss and evaluate science together. However, even such debate might be not enough to resolve all controversies, but without trying and goodwill, it is impossible to achieve a meaningful consensus.

## Author contributions

The author confirms being the sole contributor of this work and has approved it for publication.

## Footnotes

^1^ https://www.emf-portal.org/en

^2^ https://www.icnirp.org/

^3^ https://www.ices-emfsafety.org/

^4^ https://bioinitiative.org/

^5^ https://www.icems.eu/

^6^ https://icbe-emf.org/

^7^ https://www.fcc.gov/document/dc-circuit-decision-environmental-health-trust-v-fcc

^8^ https://bioinitiative.org/updated-research-summaries/

^9^ https://eur-lex.europa.eu/legal-content/EN/TXT/PDF/?uri=CELEX:52000DC0001&from=EN

^10^ https://monographs.iarc.who.int/wp-content/uploads/2019/10/IARCMonographs-AGReport-Priorities_2020-2024.pdf

^11^ https://www.gsma.com/publicpolicy/gsma_events/the-gsma-emf-forum-2022

^12^ https://roundtableinitiativeoncellphones.wordpress.com/2013/02/10/starting-the-the-round-table-initiative/

^13^ https://roundtableinitiativeoncellphones.wordpress.com/2013/02/11/formal-response-from-icnirp/

^14^ https://roundtableinitiativeoncellphones.wordpress.com/2013/02/10/response-from-the-bioinitiative-2/

^15^IARC Monographs Preamble (https://monographs.iarc.who.int/iarc-monographs-preamble-preamble-to-the-iarc-monographs/).

^16^IARC Instructions for participating scientists (https://monographs.iarc.who.int/preamble-instructions-for-authors/).
